# Radio-frequency capacitance spectroscopy of metallic nanoparticles

**DOI:** 10.1038/srep10858

**Published:** 2015-06-04

**Authors:** James C. Frake, Shinya Kano, Chiara Ciccarelli, Jonathan Griffiths, Masanori Sakamoto, Toshiharu Teranishi, Yutaka Majima, Charles G. Smith, Mark R. Buitelaar

**Affiliations:** 1 Cavendish Laboratory, University of Cambridge, Cambridge CB3 0HE, United Kingdom; 2 Materials and Structures Laboratory, Tokyo Institute of Technology, Yokohama 226-8503, Japan; 3 Institute for Chemical Research, Kyoto University, Uji 611-0011, Japan; 4 PRESTO, Japan Science and Technology Agency, Uji 611-0011, Japan; 5 London Centre for Nanotechnology, UCL, London WC1H 0AH, United Kingdom; 6 Department of Physics and Astronomy, UCL, London WC1E 6BT, United Kingdom

## Abstract

Recent years have seen great progress in our understanding of the electronic properties of nanomaterials in which at least one dimension measures less than 100 nm. However, contacting true nanometer scale materials such as individual molecules or nanoparticles remains a challenge as even state-of-the-art nanofabrication techniques such as electron-beam lithography have a resolution of a few nm at best. Here we present a fabrication and measurement technique that allows high sensitivity and high bandwidth readout of discrete quantum states of metallic nanoparticles which does not require nm resolution or precision. This is achieved by coupling the nanoparticles to resonant electrical circuits and measurement of the phase of a reflected radio-frequency signal. This requires only a single tunnel contact to the nanoparticles thus simplifying device fabrication and improving yield and reliability. The technique is demonstrated by measurements on 2.7 nm thiol coated gold nanoparticles which are shown to be in excellent quantitative agreement with theory.

Electrical characterization of metallic nanoparticles using dc transport methods requires both a source and drain electrode to pass a current as well as a gate electrode to vary the chemical potential, see Fig. 1a. The most commonly used fabrication techniques are electromigration, where a metallic wire is controllably loaded to failure such that a nanosize gap opens up in which particles might be trapped, or electrode plating in which a predefined gap is reduced electrochemically^1^,^2^,^3^. Although these methods are certainly feasible, the need for two tunnel contacts makes the fabrication complex and challenging and the quality and yield of devices highly variable. This is particularly problematic for the smallest nanoparticles - or molecules - less than around 3 nm in size.

Here we present a different technique that allows the study of arbitrarily small nanoparticles using only a single tunnel contact. Importantly, the technique does not require nm positioning accuracy which facilitates the use of standard lithography methods. As illustrated in Fig. 1b, we achieve this by placing the nanoparticles on a metal electrode and and by *capacitively* coupling the nanoparticle to a second electrode, which functions both as an ac reference ground and as a dc gate electrode. The devices are embedded in a resonant LC circuit and radio-frequency reflectometry is used as the measurement tool^4^,^5^,^6^,^7^,^8^. This approach, which can be considered as capacitance spectroscopy^9^,^10^ taken into the radio-frequency domain, not only simplifies device fabrication but also offers high sensitivity and high bandwidth readout.

The resonant circuits used in our experiments typically have a resonant frequency *f*_0_ in the 300-500 MHz range which is given by the total device capacitance *C*_Σ_ - including parasitics - and a chip inductance *L* placed on the sample holder such that 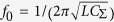, see Fig. 1d. The key idea is that the capacitance depends in part on the ability of electrons to move on and off the nanoparticle at the driving frequency which in turn depends on the chemical potential of the nanoparticle which can be tuned by the gate electrode. The gate-dependent capacitance is probed by measuring the phase shift of a reflected radio-frequency signal at the resonant frequency *f*_0_.

Before discussing experimental data, using Au nanoparticles, we will first examine, quantitatively, the expected phase response of the reflectometry technique for a generic ‘electron-in-a-box’ system or quantum dot with discrete energy spectrum, coupled to a metallic lead at some finite temperature *T*, as illustrated in Fig. 2. In the presence of a radio-frequency signal, as in Fig. 1b, the energy levels on the quantum dot will oscillate with respect to the lead. However, it is only possible for electrons to move on and off the quantum dot when an energy level is aligned with the thermally broadened electrochemical potential of the lead. In this case, the occupation probability of the level varies with the drive frequency and the movement of charge on and off the electrode in response to the rf drive can be parameterized by an effective admittance *Y* = 1/Δ*R* + *jω*Δ*C* where *ω* = 2*πf* is the radial frequency and with capacitance Δ*C* and resistance Δ*R* as shown in Fig. 2b. If the thermal energy is larger than the lifetime broadening of the resonances, *k*_*B*_*T* ≫ *hγ*, where *γ* is the tunnel rate, it is relatively straightforward, see Supporting Information, to obtain the following relations using rate equations:
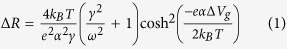

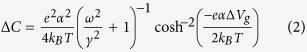
where the term −*eα*Δ*V*_*g*_ takes into account the position of the discrete energy level with respect to the Fermi level of the lead using a lever arm *α* for conversion from gate voltage to energy^6^,^11^. If the tunnel rate is comparable to the drive frequency, *γ* ~ *ω*, dissipation can be significant as previously observed in single-electron tunneling devices^12^,^13^. However, if tunnel rates are considerably faster than the drive frequency, that is *γ* ≫ *ω*, the effective resistance Δ*R* diverges and the junction will be capacitive only. It is this limit that we will consider here. Using standard network analysis^14^, it can be shown, see Supporting Information, that in this case the measured phase shift, for an under-coupled resonator, is related to the effective capacitance as:
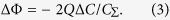
where *Q* is the quality factor of the resonator given by the ratio of the resonant frequency and bandwidth Δ*f* of the resonance. Using the relations 1-3 above we now have the tools to quantitatively investigate single-electron tunneling of any device of nm dimensions in which the energy spectrum is discrete.

## Results and Discussion

### Nanoparticle measurements

**Figure 3:**
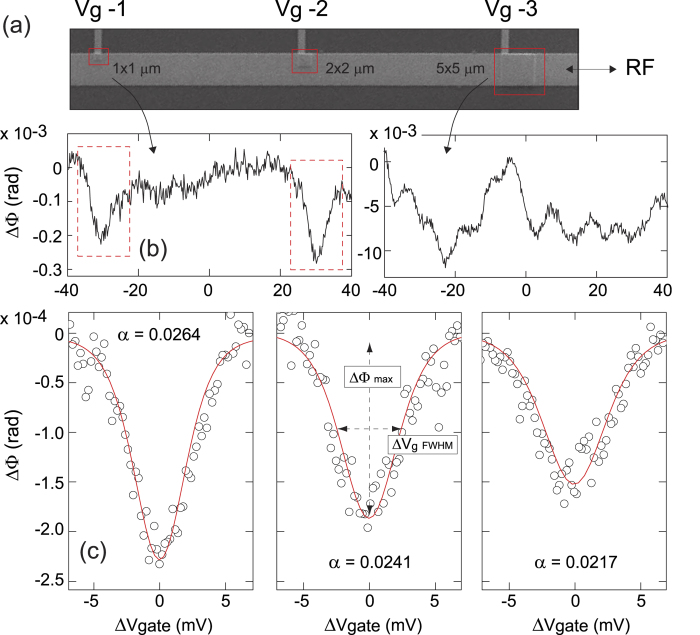
(a) Scanning electron microscope image of a nanoparticle device showing three gate electrodes with different overlap with the rf electrode. (**b**) Gate dependent phase response for a 1 × 1 *μ*m (left) and 5 × 5 *μ*m gate area (right). For the smallest gate areas fewer particles are being detected and individual resonances are observed. (**c**) Fitted curves to three individual dips observed in a small device. A constant background slope seen in all data, including control samples, has been subtracted. For the dips we obtain *α* = 0.0264, 0.0241, and 0.0217 from left to right, respectively. Since the widths of the dips scale, for a given temperature *T*, as Δ*V*_*gFWHM*_ ∝ 1/*α* and their depths as ΔΦ_*max*_ ∝ *α*^2^ the dips with lower *α* are both wider and shallower.

To demonstrate the technique experimentally, we have deposited thiol-coated Au nanoparticles of 2.7 nm diameter on a gold substrate. These particles were then covered by approximately 5.5 nm of Al_2_O_3_ deposited using atomic layer deposition, see Methods. On top of the oxide layer we defined a set of gate electrodes of varying sizes as illustrated by the scanning electron microscope (SEM) images in Fig. 3a. The results of measurements on two representative structures of different size in a He-3 cryostat with a base temperature of ~400 mK are shown in Fig. 3b. The number of particles under the large 5 × 5 *μ*m gate electrodes is not exactly known but, given an approximate density of Au nanoparticles of ~10-50 *μ*m^−2^, see Supporting Information, is likely to be large. Therefore, even though for individual particles the energy levels are expected to be well separated, the measurements on this gate electrode show an interleaving of the signal of many particles, resulting in the oscillatory pattern seen in the right panel image of Fig. 3b. For the measurements shown in the left panel of Fig. 3b, the gate overlap is significantly reduced - by a factor of 25 - and we observe more pronounced dips against a relatively flat background. For these devices the number of nanoparticles under the gate electrodes is sufficiently low for the observation of tunneling through individual electron states as we will examine in more detail. We also performed a series of control experiments, see Supporting Information, in which no nanoparticles were deposited and in which we did not see any features in the measurements.

Using the relations 2 and 3 obtained above for ΔΦ versus Δ*V*_*g*_ we are able to compare the experimental data with theory. We obtain an excellent fit for individual resonances, see Fig. 3c, where the only free parameter is the lever arm *α* which sets both the width and depth of the dips. The width of the dips Δ*V*_*g*_ ∝ *T*/*α*, or, more precisely, the full-width-half-maximum is 3.53 *k*_*B*_*T*/*eα* which is identical to what is expected in dc transport of individual quantum states^15^,^16^,^17^. However, unlike dc transport in which the magnitude of the conductance is set by the tunnel rate and asymmetry of the source and drain tunnel junctions, in the reflectometry measurements - in which there is only a single tunnel junction - the magnitude ΔΦ ∝ *α*^2^/*T*. We thus have two relations for *α* and *T* which can be obtained independently. Indeed, while in our experiments the temperature is known, *T* *~* 400 mK, if the temperature is set as an additional fit parameter, the fit procedure yields the correct experimental result. This enables an energy calibration without the need of a source-drain bias voltage and allows measurements of, for example, the charging energy of the nanoparticles or, when a magnetic field is applied, measurements of the g-factor or energy spectrum^9^,^10^.

### Nano-thermometry

A further important consequence is that our devices can be used as a primary thermometer, that is, a thermometer that allows temperature measurements without calibration by another thermometer. The advantage of using a thermometer based on nanoparticles is that it works over an exceptionally large temperature range. The lower limit of the thermometer is set by the requirement that lifetime broadening due to the coupling to the lead is less than thermal smearing: 2*hγ* < *k*_*B*_*T*. Since at the same time, the tunnel rates should be comparable to or larger than the rf driving frequency, a practical limit would be 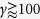 MHz which corresponds to about 10 mK. The higher limit of the thermometer is set by the spacing between adjacent dips in measurements as in Fig. 3b. For a single nanoparticle this separation is given by the addition energy which exceeds several tens of meV for small nanoparticles^18^ corresponding to room temperature operation^19^. The energy-level separation Δ*E* of Au nanoparticles of 2.7 nm diameter is of order 10 meV such that for temperatures above around 100 K a transition from the quantum (Δ*E* ≫ *k*_*B*_*T*) to the classical (Δ*E* ≪ *k*_*B*_*T*) transport regime is expected. In this case Eq. 2 is no longer valid, although the resonances will still be thermally broadened and can be modeled^16^,^17^. A further practical consideration is that the signal strength ΔΦ ∝ *α*^2^/*T*, and thus decreases with increasing temperature. This can be compensated for by increasing the lever arm *α* which is set by the gate oxide thickness and dielectric constant. Using Al_2_O_3_, we found ~5 nm to be the optimal oxide thickness for our devices: thicker oxides reduced the signal-to-noise ratio while thinner oxides affected device yield and resulted in breakthrough when the gate voltage was increased beyond ~0.5 V. Nevertheless, the lever arm can be increased significantly by using high-dielectric oxides such as HfO_2_ or TiO_2_ which would improve signal-to-noise ratios by about two orders of magnitude. By a further improvement of the quality factor of the resonator, for example by reducing parasitic capacitances, we believe that room temperature operation is feasible. By embedding the device in a resonant circuit, as in this work, measurements are also fast and not limited by 1/f noise as in conventional dc quantum dot thermometry^20^,^21^.

The experiments on Au nanoparticles described here are readily extended to other types of nanoparticles such as Pt, Fe, or Co nanoparticles, which have applications in a range of areas varying from catalysis to biomedicine^22^,^23^, single molecule magnets such as Mn_12_, Cr_7_Ni or TbPc_2_, or endohedral fullerenes which are of interest in spintronics or spin-based quantum information processing^24^,^25^,^26^. As identified above, there is furthermore significant scope for improvement in the measurement sensitivity - and thus the operating temperature - of the nanodevices by optimization of the oxide dielectrics and quality factor of the resonators used. Chemical modification of the length of linker molecules, such as decanedithiol used in this work, provides control over the tunnel rates while varying the nanoparticle solution pH and adsorption time^27^, and surface modification of the electrodes^28^,^29^,^30^ - or a combination of these - allows nanoparticle density control. Taken together, this opens up novel routes for the characterization and application of individual molecules and metallic nanoparticles.

## Methods

Devices were fabricated on nominally undoped Si wafer substrates of resistivity >100 Ohm cm patterned with Ti/Au electrodes using electron-beam lithography. Before deposition of the nanoparticles, the electrodes were cleaned using acetone, ethanol and an oxygen plasma, followed by a further ethanol immersion to remove any surface oxides. The devices were then immersed in a toluene-based gold nanoparticle solution for one hour, followed by a rinse with toluene and ethanol. The gold nanoparticles have a 2.7 nm core diameter and were coated with octanethiol (length: 1.44 nm) and decanedithiol (1.82 nm) mixed monolayer. The devices were subsequently covered by approximately 5.5 nm of Al_2_O_3_ using atomic layer deposition. In the final stage, a further set of Ti/Au gate electrodes were defined on the devices using electron-beam lithography. The devices typically showed no leakage between electrodes separated by the Al_2_O_3_ layer up to around 0.5 V, at which point the oxide coating on the devices began to break down.

The devices were mounted on a microstrip line coated printed circuit board sample holder with radio-frequency connections. Measurements were carried out at *T* *~* 400 mK in a Helium-3 cryostat, custom modified to allow reflectometry radio-frequency measurements, with a low noise cryogenic amplifier to improve the signal-to-noise ratio of the reflected signal. Demodulation is achieved by mixing the reflected rf signal with the reference signal. Both quadratures of the signal are detected, allowing measurements of both the amplitude and phase response. For the measurements shown in Fig. 3 a chip inductor *L* = 500 nH was used. This yielded a resonance frequency *f*_0_ = 345 MHz and capacitance *C*_Σ_ = 0.43 pF with quality factor Q ~ 59 which were the parameters used in the fit procedure for Fig. 3c. The dc gate voltage was applied to the samples via a bias-tee.

## Additional Information

**How to cite this article**: Frake, J. C. *et al.* Radio-frequency capacitance spectroscopy of metallic nanoparticles. *Sci. Rep.* **5**, 10858; doi: 10.1038/srep10858 (2015).

## Supplementary Material

Supplementary Information

## Figures and Tables

**Figure 1:**
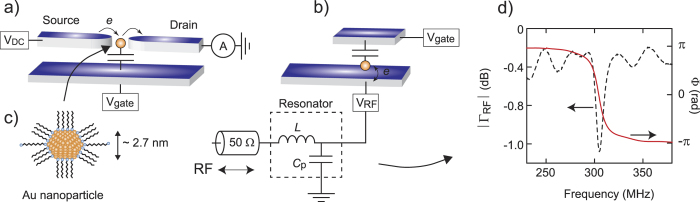
(a) Schematic of a conventional direct-current measurement.Two transport electrodes that are precisely aligned and separated by a few nanometers are required to enable electron transport through a nanoparticle. (**b**) Schematic of the radio-frequency reflectometry technique. The nanoparticle is only tunnel coupled to a *single* electrode. A second electrode, which does not need to be precisely aligned, functions both as an ac ground and dc gate electrode. The device is embedded in a LC resonant circuit which both simplifies device fabrication and allows for high sensitivity and high bandwidth measurements. (**c**) Representation of a gold nanoparticle coated in thiol chains used in our experiments. (**d**) Measured amplitude and phase response of a resonant circuit with resonant frequency ~305 MHz.

**Figure 2:**
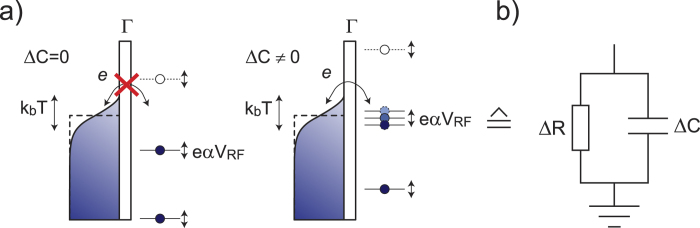
(a) Schematic energy diagrams showing the discrete energy states of the nanoparticle and the temperature broadened electron distribution in the lead. The occupation probabilities of the states on the nanoparticle depend on the relative position of the states with respect to the electrochemical potential of the lead and varies from fully occupied (filled circles) to unoccupied (open circles). In response to the rf excitation, the relatively position of the levels oscillate at the applied frequency. Left: without a state in the thermal window around the electrochemical potential of the lead, no tunneling is allowed: all states are either fully occupied or empty. Right: when a level is aligned with the electrochemical potential of the lead, the average occupancy of the state oscillates at the driving frequency and charge thus moves back and forth between the particle and the lead (**b**) The device can be parameterized by an effective RC circuit as explained in the main text.
