# Supplementary material for: Radio-frequency capacitance spectroscopy of metallic nanoparticles
Source: Sci Rep. 2015 Jun 4;5:10858. doi: 10.1038/srep10858 (PMC4650672; doi:10.1038/srep10858)
Supplement: Supplementary Information [file srep10858-s1.pdf]

Radio-frequency capacitance spectroscopy of metallic nanoparticles: Supplementary Material

J.C. Frake,[1] S. Kano,[2,3] C. Ciccarelli,[1] J. Griffiths,[1] M. Sakamoto, [3,4,5]
T. Teranishi, [3,4] Y. Majima,[2,3,6] C. G. Smith,[1] and M. R. Buitelaar[7,8]

[1] *Cavendish Laboratory, University of Cambridge, Cambridge CB3 0HE, United Kingdom*
[2] *Materials and Structures Laboratory, Tokyo Institute of Technology, Yokohama 226-8503, Japan*
[3] *CREST, Japan Science and Technology Agency, Yokohama 226-8503, Japan*
[4] *Institute for Chemical Research, Kyoto University, Uji 611-0011, Japan*
[5] *PRESTO, Japan Science and Technology Agency, Uji 611-0011, Japan*
[6] *Department of Printed Electronics Engineering, Sunchon National University, Sunchon 540-742, South Korea*
[7] *London Centre for Nanotechnology, UCL, London WC1H 0AH, United Kingdom*
[8] *Department of Physics and Astronomy, UCL, London WC1E 6BT, United Kingdom*

The following sections comprise the supplementary material to the main text. We provide the following: (i) An analysis of the RLC network used in the experiments; (ii) A derivation of the effective admittance of the nanoparticle devices, starting with the rate equations and (iii) The procedure used for the data fits and control experiments data in which no Au nanoparticles were present.

I RLC NETWORK

A diagram of the circuit used in this work is shown in Fig. S1. The circuit consists of a capacitance C_p which takes into account the parasitics of the components and wiring, an inductor L which consists of the chip inductor placed on the sample holder, and a resistor R_m which takes into account the resistances in the wiring and the inductor - but could also double to allow for a matching resistor. The nanoparticle device, when modeled as a lumped component resistor ΔR and capacitor ΔC in parallel, as described in Section II below, allows the treatment of the entire system as a simple tank circuit. The impedance of the resonant circuit can then be described as:

$$Z_{tot} = R_m + i\omega L + \Delta R \parallel (\Delta C + C_p) \tag{1}$$

Expanding out the terms and arranging into real and imaginary components, we arrive at the impedance of the circuit as a function of the 'components' of the device, ΔR and ΔC.

$$Z_{tot} = \left[R_m + \frac{\Delta R}{1 + (\omega C_\Sigma \Delta R)^2}\right] + i\omega \left[L - \frac{C_\Sigma \Delta R^2}{1 + (\omega C_\Sigma \Delta R)^2}\right] \tag{2}$$

where $C_\Sigma = C_p + \Delta C$. From here, it is possible to understand how a shift in the resistance or capacitance of the

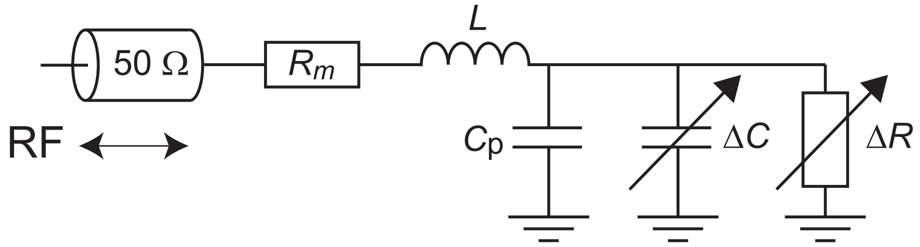

Figure S 1: Schematic model of the RLC circuit used in the radio-frequency reflectometry measurements. The nanoparticle device is modeled as a lumped component resistor ΔR and capacitor ΔC in parallel which allows the treatment of the entire system as a simple tank circuit.

device will change the response seen on the circuit. We can simplify this equation by looking at how it behaves near resonance, since this is the nominal operation range - as carried out in the resonant circuit model of Ref. [1]. Making the (generally good) approximation that $\omega\Delta RC_\Sigma >> 1$ and substituting $\omega_0^2 = 1/(LC_\Sigma)$:

$$Z_{tot,res} = R_m + \frac{L}{\Delta RC_\Sigma} + i\omega L - \frac{i}{\omega C_\Sigma} \tag{3}$$

where the imaginary component vanishes to zero exactly on resonance. Note that in our work ΔR is typically very large, which differs from the work by, e.g., Schoelkopf et al [2] where the resonant circuit is used to down-convert the resistance of a single-electron transistor (typically of order 10 kΩ) to 50 Ω. From here on, unless explicitly stated, the frequency is assumed to be on resonance. If we define Z_r and Z_i as the real and imaginary components of the impedance of the circuit, and Z_0 as the $50\,\Omega$ impedance of the wave guide connected to the tank circuit, then the reflection coefficient is given by:

$$\Gamma = \frac{Z - Z_0}{Z + Z_0} = \frac{Z_r^2 + Z_i^2 - Z_0^2 + 2iZ_0Z_i}{(Z_r + Z_0)^2 + Z_i^2} \tag{4}$$

This, along with the calculated impedance from above, can be put together to show how the measured response of the tank circuit will vary as a function of effective dissipative ΔR and reactive ΔC components of the device, which can be in turn matched to the physics of the device itself using appropriate models.

The amplitude and phase of the reflected signal are given by:

$$|\Gamma| = \frac{\sqrt{(Z_r^2 + Z_i^2 - Z_0^2)^2 + (2Z_0Z_i)^2}}{(Z_r + Z_0)^2 + Z_i^2} \tag{5}$$

$$\Gamma_\phi = \mathrm{atan}\frac{2Z_0Z_i}{|Z|^2 - Z_0^2} \tag{6}$$

The phase response is a clear indicator of under $[Z(\omega_{res}) > 50\Omega]$ or over $[Z(\omega_{res}) < 50\Omega]$ coupling. In the latter case, the phase change through resonance will be $0 \leq \phi \leq \pi$ and the former will result in a phase shift of $\phi = 2\pi$. It is now possible to see how the magnitude and phase of the reflection coefficient vary with relevant factors. For the capacitance shift in phase of the reflection coefficient:

$$\left.\frac{\partial\Gamma_\phi}{\partial\Delta C}\right|_{\omega=\omega_0} = \frac{2Z_0}{\omega C_\Sigma^2(R_{eff}^2 - Z_0^2)} = \frac{2QZ_0}{C_\Sigma(R_{eff} - Z_0)}, \tag{7}$$

where $R_{eff} = R_m + L/(\Delta RC_\Sigma)$ and $Q = \sqrt{\frac{L}{C_\Sigma}}/(R_{eff} + Z_0)$ is the loaded quality factor of the resonator. In the limit where $R_m \to 0$ and $\Delta R \to \infty$ this reduces to Eq. 3 in the main text. For the change in reflection magnitude due to a device resistance shift (still assuming $\omega\Delta RC_\Sigma >> 1$) :

$$\left.\frac{\partial|\Gamma|}{\partial\Delta R}\right|_{\omega=\omega_0} = \frac{2LZ_0}{C_\Sigma\Delta R^2(R_{eff} + Z_0)^2} = \frac{2Z_0Q^2}{\Delta R^2}. \tag{8}$$

And finally, the change in magnitude due to a capacitance shift:

$$\left.\frac{\partial|\Gamma|}{\partial\Delta C}\right|_{\omega=\omega_0} = \frac{-2Z_0Q^2}{C_\Sigma\Delta R}. \tag{9}$$

From this, it can be seen that for a fixed device capacitance, the magnitude shift becomes far more sensitive to resistive changes for a device with smaller resistance. It should also be said that the change of reflection coefficient angle is negligible for changes of device resistance in the case where ΔR is large.

II EFFECTIVE ADMITTANCE: RATE EQUATIONS

To calculate the effective admittance of the nanoparticle device we start by assuming that we are in the incoherent regime such that the tunnel coupling γ is sufficiently small: $h\gamma \ll k_b T$. The tunnel rates for a single electron to tunnel off and on a level at energy ϵ relative to the Fermi energy of the tunnel electrode are given by [3, 4]:

$$\gamma_{off} = \gamma(1 - f(\epsilon)) \tag{10}$$
$$\gamma_{on} = \eta\gamma f(\epsilon) \tag{11}$$

where $\eta = 2$ indicates spin degeneracy and $f(\epsilon)$ is a Fermi function. To simplify the analysis we will use $\eta = 1$ here which does not affect the results - apart from a temperature-dependent shift of the resonance center [5] which is not important for the analysis below. In the presence of an rf drive, the position of the level relative to the Fermi energy of the tunnel electrode is given by:

$$\epsilon(t) = -e\alpha(V_g + V_{RF}\mathrm{e}^{i\omega t}) \tag{12}$$

where we make use of the lever arm $\alpha = C_R/(C_L + C_R)$ where C_R and C_L are the geometric capacitances between the nanoparticle and the gate electrode and the nanoparticle and the rf electrode, respectively. Typically for our devices $C_R \ll C_L$ and as a result $\alpha \ll 1$. The probabilities for the level to be either empty (P_0) or occupied (P_1) by a single charge are:

$$\frac{\mathrm{d}P_0}{\mathrm{d}t} = \gamma_{off}P_1 - \gamma_{on}P_0 \tag{13}$$
$$\frac{\mathrm{d}P_1}{\mathrm{d}t} = \gamma_{on}P_0 - \gamma_{off}P_1 \tag{14}$$

Since $P_0 + P_1 = 1$ we arrive at the master rate equation:

$$\frac{\mathrm{d}P_1}{\mathrm{d}t} + \gamma P_1 = \gamma f(\epsilon) \approx \gamma\left(1 + \left(1 - \frac{e\alpha V_{RF}}{k_B T}\mathrm{e}^{i\omega t}\right)\mathrm{e}^{\frac{-e\alpha V_g}{k_B T}}\right)^{-1} \tag{15}$$

where we use a small excitation approximation, i.e. $e\alpha V_{RF} \ll k_B T$. Notice that the gate voltage term cannot be assumed small in comparison with thermal energies. Using $G = \mathrm{e}^{-(e\alpha V_g)/(k_B T)}$ for brevity, the solution to this differential equation is a hypergeometric function [6]:

$$P_1 = \left(\frac{1}{1+G}\right)\,{}_2\mathrm{F}_1\left(1, -\frac{i\gamma}{\omega}; 1 - \frac{i\gamma}{\omega}; \frac{G}{1+G}\frac{e\alpha V_{RF}\mathrm{e}^{i\omega t}}{k_B T}\right) \tag{16}$$

This can be expressed as a sum:

$$P_1 = \frac{1}{1+G}\left[1 + \sum_{n=1}^{\infty}\frac{-i\gamma}{\omega n!(n - \frac{i\gamma}{\omega})}\left(\frac{G}{1+G}\frac{e\alpha V_{RF}\mathrm{e}^{i\omega t}}{k_B T}\right)^n\right] \tag{17}$$

This is the full response of a single electron revoir with a single lead and single gate to a small oscillation applied to the source. The terms in this sum die off rapidly with increasing n, so by taking the first term ($n = 1$), we can extract much of the physics of the system without too much complexity.

$$P_1 \approx \frac{1}{1+G}\left[1 - \frac{\frac{i\gamma}{\omega}}{1 - \frac{i\gamma}{\omega}}\frac{G}{1+G}\frac{e\alpha V_{RF}\mathrm{e}^{i\omega t}}{k_B T}\right] \tag{18}$$

The expected charge drawn from a voltage source connected to the RF electrode at time t is given by $Q_{RF}(t) = e\alpha P_1(t)$. The expected current flowing from the voltage source is then:

$$i_{RF}(t) = \frac{dQ_{RF}(t)}{dt} = \frac{G}{(1+G)^2}\frac{\omega\gamma}{\omega - i\gamma}\frac{e^2\alpha^2}{k_BT}V_{RF}\mathrm{e}^{i\omega t} \tag{19}$$

The effective impedance is thus:

$$Z_{eff} = V_{RF}e^{i\omega t}/i_{RF} = \frac{(1+G)^2}{G}\frac{k_BT}{e^2\alpha^2}\frac{\omega^2+\gamma^2}{\omega\gamma(\omega+i\gamma)} \tag{20}$$

Equating this with $Z_{eff}^{-1} = R_{eff}^{-1} + j\omega C_{eff}$ we find that:

$$R_{eff} = \frac{k_BT}{e^2\alpha^2\gamma}\left(\frac{\gamma^2}{\omega^2}+1\right)\frac{(1+G)^2}{G} \tag{21}$$

$$C_{eff} = \frac{e^2\alpha^2}{k_BT}\left(\frac{\omega^2}{\gamma^2}+1\right)^{-1}\frac{G}{(1+G)^2} \tag{22}$$

Identifying $(1+G)^2/G$ as $4\cosh^2\left(\frac{-e\alpha\Delta V_g}{2k_BT}\right)$ we arrive at Eq. 1 and 2 in the main text.

III DATA ANALYSIS AND CONTROL DEVICES

Using Eq. 7 and Eq. 22 (or Eq. 2 and Eq. 3 in the main text) we are able to fit the measured data to theory, provided C_Σ and Q are known. We obtain both from the measured amplitude and phase response over a large frequency range which shows a resonance at $f = 345$ MHz. Knowing the value of the chip inductor $L = 500$ nH this yields $C_\Sigma = 0.43$ pF. The quality factor is measured by fitting the phase response to: $\phi(f) = \phi_0 + 2\,\mathrm{atan}[2Q(1 - f/f_0)]$ where ϕ_0 is the angle at the resonance frequency f_0. This yielded a best fit to the data of $Q = 59 \pm 4$. This is somewhat larger than expected for a $Z_0 = 50\ \Omega$ loaded circuit which we tentatively attribute to an impedance mismatch at the cryogenic amplifier connected to the circuit. For the data fits in the main text we used $\gamma \gg \omega$ and the experimentally determined $C_\Sigma = 0.43$ pF and $Q = 59$. A constant background slope, seen in all data including control samples, see e.g. Fig. S2b below, has been subtracted.

The full-width-half-maximum (FWHM) of the resonances as a function of gate voltage (V_g) described by Eq. 22 provides a relation between temperature and lever arm:

$$\Delta V_{g,FWHM} = \frac{k_BT}{e\alpha}(ln(3+\sqrt{8}) - ln(3-\sqrt{8})) \approx 3.53\frac{k_BT}{e\alpha} \tag{23}$$

The gate dependency is the differential of the fermi function, as expected. Using Eq. 7 and 22, the maximum phase signal is given by:

$$|\Delta\Phi_{max}| = \frac{2Q}{C_\Sigma}\frac{e^2\alpha^2}{4k_BT} \tag{24}$$

From these relations we can determine the temperature and lever arm as:

$$T = \frac{2C_\Sigma|\Delta\Phi_{max}|\Delta V_{g,FWHM}^2}{(3.53)^2 k_BQ} \qquad \alpha = \frac{2C_\Sigma|\Delta\Phi_{max}|\Delta V_{g,FWHM}}{3.53eQ} \tag{25}$$

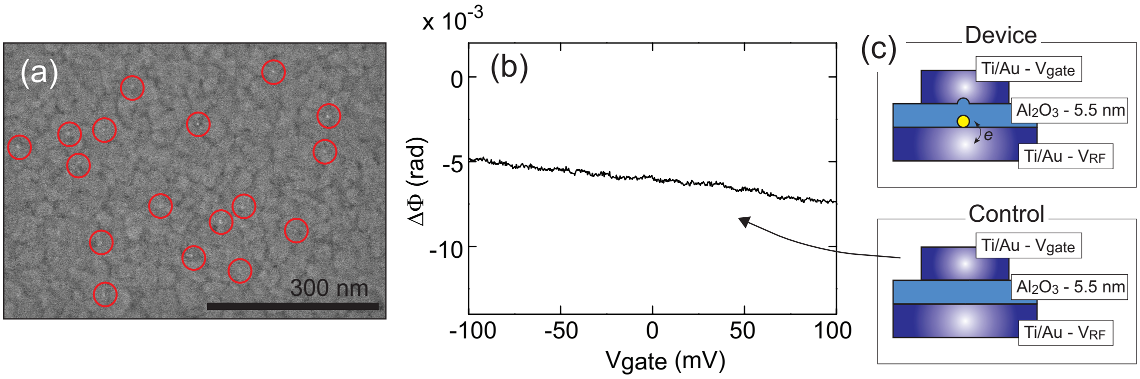

Figure S 2: **(a)** Scanning electron micrograph of a device *with* Au nanoparticles as indicated by the red circles. **(b)** Phase data from a control device without nanoparticles for large 5x5 μm gate area. The vertical scale bar (phase) is set to allow for a direct comparison with Fig. 3b in the main text. **(c)** Schematic of the nanoparticle (top) and control (bottom) devices.

We are thus able to determine the temperature from measurements of the peak width and height directly which does not require a separate measurement of the lever arm. The signal strength is maximized by improving the lever arm and quality factor. As described in the main text, the devices can be used as a primary thermometer, the limits of which are set by the tunnel coupling γ and the 0D level spacing and charging energies of the nanoparticles.

To ensure that we measured the nanoparticles and not some other effect, e.g. charge traps in the oxides, control samples were prepared. When nanoparticles are deposited, a rough indication of the average density is obtained from scanning electron micrographs, see Fig. S2a. For the control devices, exactly the same fabrication procedure as outlined in the Methods section in the main text was carried out, with the exception that no nanoparticles were deposited onto the substrates. All other variables were kept the same, and the various control devices measured. An example is shown in Fig. S2b, for which indeed no signal is observed apart from a slowly decreasing background seen in all devices - leading to the conclusion that the nanoparticles are indeed responsible for resonances in the data. Samples *with* Au nanoparticles but with considerable thicker gate oxides ($>$ 10 nm Al_2O_3) also did not show a response at 400 mK (not shown) as expected since for these devices the lever arm will be much smaller and thus the measured signal strength, as expressed by Eq. 24.

---

[1] Roschier, L. *et al.* Noise performance of the radio-frequency single-electron transistor, Journal of Applied Physics 95, 1274 (2004).
[2] Schoelkopf, R.J., Wahlgren, P., Kozhevnikov, A.A., Delsing, P. and Prober, D.E. The radio-frequency single-electron transistor (RF-SET): A fast and ultrasensitive electrometer. Science 280, 1238 (1998).
[3] MacLean, K. Energy-dependent tunneling in a quantum dot. Phys. Rev. Lett. 98, 036802 (2007).
[4] Petersson, K.D., PhD thesis: High frequency manipulation and measurement of charge and spin states in GaAs quantum dot devices, Cambridge (UK), 2009.
[5] Bonet, E. Deshmukh, M.M. and Ralph, D.C. Solving rate equations for electron tunneling via discrete quantum states, Phys. Rev. B 65, 045317 (2002).
[6] Arfken, G.B and Weber, H.J. Mathematical methods for physicists, 6ed edition, Elsevier Academic Press (2005).
